# Mechanized transplanting with side deep fertilization increases yield and nitrogen use efficiency of rice in Eastern China

**DOI:** 10.1038/s41598-019-42039-7

**Published:** 2019-04-04

**Authors:** Conghua Zhu, Jing Xiang, Yuping Zhang, Yikai Zhang, Defeng Zhu, Huizhe Chen

**Affiliations:** 1 State Key Laboratory of Rice Biology, China National Rice Research Institute, Hangzhou, 311400 P.R. China; 2 Crop Research Institute, Sichuan Academy of Agricultural Sciences, Chengdu, 610066 P.R. China

## Abstract

The application of nitrogen (N) fertilizer deep in soil at the same time as mechanical transplanting of rice seedlings is an effective alternative to conventional broadcasting of fertilizer, but its effects on yields and profitability have not been analysed in detail. Here, we analysed the effects of a side deep application of N fertilizer at transplanting on the N uptake, N use efficiency (NUE), grain yield, and economic profitability of two rice (*Oryza sativa* L.) cultivars; Yongxian15 (early season) and Yongyou1540 (middle/late season). In the field experiments, two types of N fertilizer (urea (U) and controlled-release urea (CRU)) were surface broadcasted manually (B) or mechanically fertilized at 5.5 ± 0.5 cm soil depth (M) (UB, UM, and CRUM treatments, respectively). The blank control had no N fertilizer (N0). Each N-fertilizer treatment had similar effects on N uptake, grain yield, NUE, and economic profitability in the early, middle, and late seasons. Compared with manually applied fertilizer, mechanically applied fertilizer increased grain yield and NUE in both cultivars. In Yongxian15 and Yongyou1540, the mechanical side deep application of N-fertilizer increased the N recovery efficiency by 62.50–91.57% and 24.38–64.24%, respectively, the N agronomy efficiency by 33.65–63.14% and 22.64–44.70%, respectively; and the grain yield by 6.30–11.64% and 6.23–13.11%, respectively. The CRUM treatments had the highest benefit–cost ratio because of high gross returns and low fertilization costs. The mechanized side deep application of N fertilizer can increase the efficiency and profitability of rice production.

## Introduction

China is not only a major rice-growing country, but also the largest rice consumer in the world. China’s grain supply and demand strongly affect the security of grain supply locally and in other countries^1–3^. With progresses in socioeconomic and technological development, labour shortages have arisen in Chinese agriculture. Therefore, there is a need to mechanize the whole process of rice production to increase the economic benefits of rice cultivation^4–8^. In conventional mechanized transplanting, equal row transplanting is the most commonly used method, and basal fertilizer is mainly broadcasted onto the soil surface manually. Therefore, fertilization is the weak point in equal row transplanting^8–10^, because it not only reduces fertilization uniformity, but also increases labour costs, resulting in lower profits and net income for rice growers. Increasing the degree of mechanization in rice fertilization will solve these problems by improving uniformity and reducing labour costs, thus increasing the efficiency of rice production.

Factors that have been shown to affect the grain yield and nitrogen (N)-use efficiency (NUE) of rice include the N fertilizer application rate^11,12^, deep application of N fertilizer^13–17^, N fertilizer application strategy^18^, balanced fertilization^19^, water management^20^, biochemical inhibitors^21^, the type of N fertilizer^20,22,23^, and real-time and on-site N fertilizer management^24^. Those studies showed that the type of fertilizer and method of fertilization were the two most important factors in grain yield and NUE. Urea (U) and controlled-release urea (CRU) are two major types of N fertilizers that affect the N nutrition and yield formation of rice^20,22^. A very small proportion of the N fertilizer applied to the soil is absorbed by rice seedlings, while the major portion is left unused^15,25^. The use of CRU reduces the number of fertilizer applications required and decreases N losses, thereby increasing the NUE^20,22^. Split fertilization with CRU can stabilize rice yields, reduce ammonia volatilization from paddy fields, and increase the NUE^26^. Single fertilization with CRU can satisfy the N requirements of rice during its whole growth period, and increase available N in soil and the N content in seedlings, thereby promoting photosynthesis^27,28^. Slow- or controlled-release N fertilizers have been shown to enhance the root growth of rice, prolong the period of N release, increase N availability in the soil, promote N uptake by rice plants, and increase NUE^20,22,23^. Surface fertilization is the least effective method, and often results in considerable N losses^14^. Fertilization in shallow soil can promote root growth in the shallow layer, which favours seedling growth^29,30^. Deep fertilization can deliver nutrients precisely to the root zone, which reduces nutrient losses, enhances N uptake by seedlings, and increases NUE and grain yield^15,16,31–33^. Deep application of U at a soil depth of 10–15 cm can effectively reduce the release of N_2_O and NO^15^, which also reduces N losses by run-off^34^, while increasing NUE^14^ and rice yield^35,36^. Compared with split fertilization, which is a common practice among farmers, single N fertilization to the root zone was shown to prolong the retention of nutrients in soil, and decrease the N loss rate from 73.0% to 29.7%, which in turn increased the apparent NUE by 22.6–30.6%^25^. Deep placement of U and compound fertilizer either alone or in combination can increase the photosynthetic rate of directly sown rice, delay ageing, and increase NUE and rice yield, as well as increasing economic benefits^37^.

**Figure 1:**
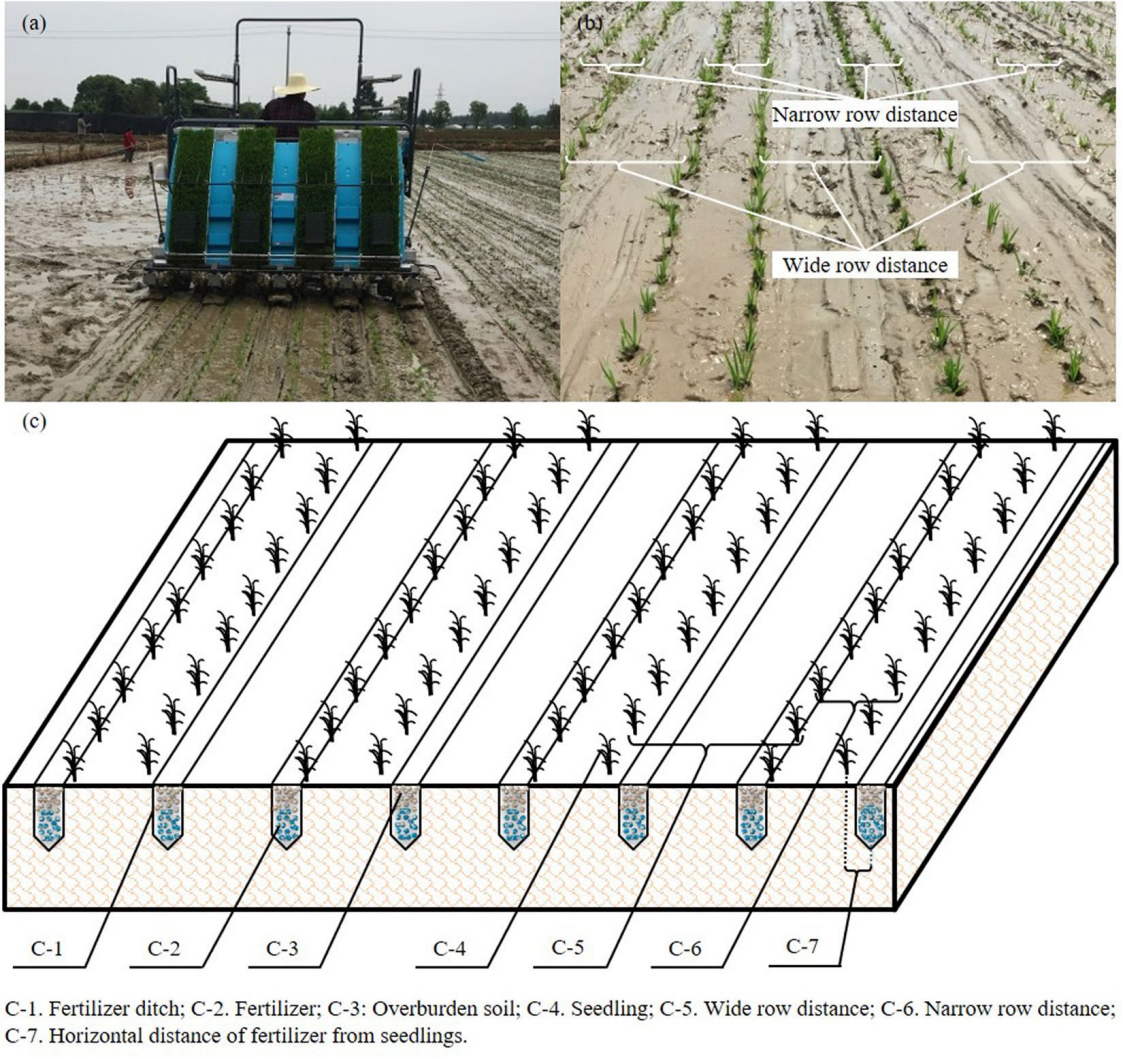
(**a**) Mechanized wide-narrow row transplanting of rice accompanied by side deep fertilization of nitrogen fertilizer at the Experimental Research Farm, Langxin Food Professional Cooperatives, Langya Town, Jinhua City, Zhejiang Province, China. Rice was planted in early rice season, middle rice season and late rice season in 2017. (**b**) Wide row and narrow row spacing of seedlings when transplanting. (**c**) Synchronous side deep fertilization technology within wide-narrow row transplanted rice. The horizontal distance of the mechanized deep placement of N fertilizer from the seedlings was 5.5 ± 0.5 cm.

Side deep fertilization synchronized with wide-narrow row transplanting is an emerging technique for transplanted rice that can replace conventional fertilization and equal row transplanting. This new method delivers fertilizer in more precise amounts and positions. The wide-narrow row side deep fertilizing rice transplanting machine (Fig. 1a,b) used in our field experiments delivered a uniform amount of fertilizer to a relative depth of 5.5 ± 0.5 cm (Fig. 1c). At that depth, there is a special anaerobic microenvironment that determines nutrient supply to the roots. Moreover, wide-narrow row spacing cultivation has been shown to improve ventilation and light transmission, and reduce the occurrence of pests and diseases in paddy fields. Although the mechanical transplanting of rice has been conducted widely in China and elsewhere, the effects of this method combined with side deep fertilization on grain yield, NUE, and economic profitability have not been well studied. Therefore, the main aims of the three field experiments conducted in 2017 were as follows: (1) to determine the effects of mechanized side deep fertilization on the N-uptake and NUE of machine-transplanted rice; (2) to analyse the effect of mechanized side deep fertilization on grain yield and yield components of two rice cultivars (Yongxian15 (early season) and Yongyou1540 (middle/late season)); and (3) to estimate the additional economic profitability of mechanized side deep fertilization compared with manual fertilization.

## Results

### Grain yield and yield components

In the field trials, we applied CRU and U by machine at transplanting (CRUM and UM treatments, respectively) or by manual broadcasting (UB). The blank control (N0) had no N fertilizer. The N application method and N fertilizer type greatly affected the grain yield and its components in Yongxian15 in the early season, and in Yongyou1540 in the middle and late seasons in 2017 (Table 1). In all treatments, the highest yield was in CRUM, followed by UM and UB, and the lowest grain yield was in N0. Compared with UB, UM resulted in more productive panicles and a higher grain number per panicle in all seasons. However, there was no significant difference between UM and CRUM in the number of productive panicles in Yongxian15 in the early season, and in the number of grains per panicle in Yongyou1540 in middle and late seasons. The grain yield was higher in UM than in UB in all three seasons because of higher numbers of productive panicles and grains per panicle. The 1000-grain weight of Yongyou1540 was higher in CRUM in the middle season, and in UM in the late season than in all of the UB treatments.

**Table 1:** Effects of mechanized side deep nitrogen fertilization on yield and yield components of rice in early, middle, and late seasons.

Treatments		Yield (t ha^−1^)	Productive panicle (10^4^ ha^−1^)	Grain number per panicle	Grain filling (%)	1000-grain weight (g)	Harvest index
ES	*Yongxian15*						
	N0	5.96 ± 0.24d	365.56 ± 11.43c	86.95 ± 3.45d	76.39 ± 1.63a	25.57 ± 0.44a	0.54 ± 0.03a
	UB	7.30 ± 0.51c	406.42 ± 6.20b	97.54 ± 3.11c	76.05 ± 2.38a	24.63 ± 0.43ab	0.52 ± 0.04b
	UM	7.76 ± 0.43b	427.46 ± 10.02a	102.97 ± 2.46b	74.40 ± 3.16a	24.42 ± 0.71b	0.50 ± 0.03b
	CRUM	8.15 ± 0.58a	432.41 ± 7.40a	108.20 ± 2.10a	74.43 ± 2.92a	24.54 ± 0.93b	0.50 ± 0.04b
	mean	7.29	407.96	98.92	75.32	24.79	0.51
MS	*Yongyou1540*						
	N0	7.56 ± 0.15d	183.33 ± 4.62d	222.96 ± 1.92c	88.08 ± 0.56a	23.26 ± 0.50c	0.57 ± 0.01a
	UB	10.43 ± 0.31c	218.33 ± 5.51c	239.84 ± 1.84b	86.28 ± 0.94b	23.60 ± 0.46bc	0.57 ± 0.02a
	UM	11.08 ± 0.40b	229.33 ± 5.13b	246.04 ± 4.37a	86.22 ± 1.41b	24.16 ± 0.28ab	0.57 ± 0.02a
	CRUM	11.75 ± 0.28a	245.67 ± 5.86a	246.31 ± 4.08a	83.51 ± 0.17c	24.56 ± 0.26a	0.55 ± 0.01b
	mean	10.20	219.17	238.79	86.02	23.89	0.57
LS	*Yongyou1540*						
	N0	6.16 ± 0.07d	160.00 ± 10.67d	202.65 ± 3.05c	82.68 ± 1.04a	24.83 ± 0.48ab	0.59 ± 0.02a
	UB	8.69 ± 0.07c	206.33 ± 3.51c	216.95 ± 2.37b	81.28 ± 1.60a	24.51 ± 0.45b	0.58 ± 0.02a
	UM	9.38 ± 0.07b	221.18 ± 6.88b	228.04 ± 2.17a	81.02 ± 2.38a	25.19 ± 0.22a	0.58 ± 0.01a
	CRUM	9.83 ± 0.12a	238.14 ± 8.48a	233.28 ± 4.37a	78.65 ± 0.61b	24.95 ± 0.03ab	0.58 ± 0.01a
	mean	8.52	206.41	220.23	80.91	24.87	0.58

ES, MS and LS are early, middle and late season, respectively. Values are means ± SE of three plots (three replicates). Different letters indicate significant difference among N application treatments in a season (p < 0.05, LSD multiple test).

### Leaf area index (LAI) and net photosynthesis (*P*n)

**Figure 2:**
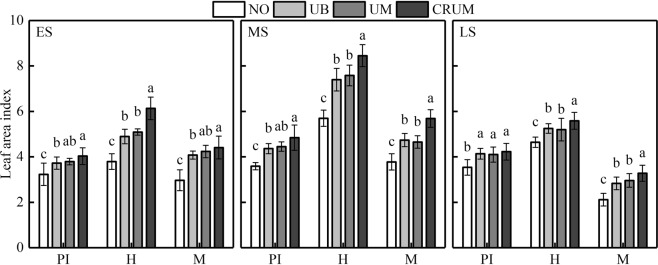
Effects of mechanized side deep nitrogen fertilization on leaf area index (LAI) of rice in early, middle, and late seasons. ES, MS and LS are early, middle and late season, respectively. PI, H and M are panicle initiation stage, heading stage and maturity stage, respectively. LAI values are means of three plots (three replicates) of Yongxian15 (ES) and Yongyou1540 (MS and LS). Within the same stage of a cultivar, different letters on columns indicate significant difference (*p* < 0.05, LSD multiple test).

**Figure 3:**
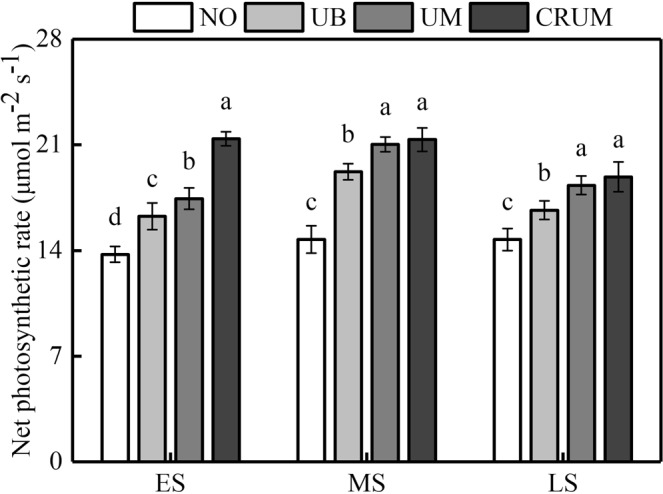
Effects of mechanized side deep nitrogen fertilization on net photosynthesis of flag leaves of rice at heading stage in early, middle, and late seasons. ES, MS and LS are early, middle and late season, respectively. Values are means ± SE of three plots (three replicates). Different letters on columns indicate significant difference (*p* < 0.05, LSD multiple test).

The LAI showed similar trends among all the treatments. The treatments could be ranked, from highest LAI to lowest, as follows: CRUM > UM > UB > N0 (Fig. 2). At the panicle initiation, heading, and maturity stages, there were no significant differences in LAI between UB and UM in all three seasons. At the heading stage, the highest LAI was in CRUM in all three seasons. Pn of Yongxian15 and Yongyou1540 at the heading stage was highest in CRUM, followed by UM and UB (Fig. 3). The treatments could be ranked, from highest Pn to lowest, as follows: CRUM > UM > UB > N0. The Pn of Yongxian15 in the early season and Yongyou1540 in the middle and late seasons differed significantly between UB and UM.

### Total above-ground biomass

**Figure 4:**
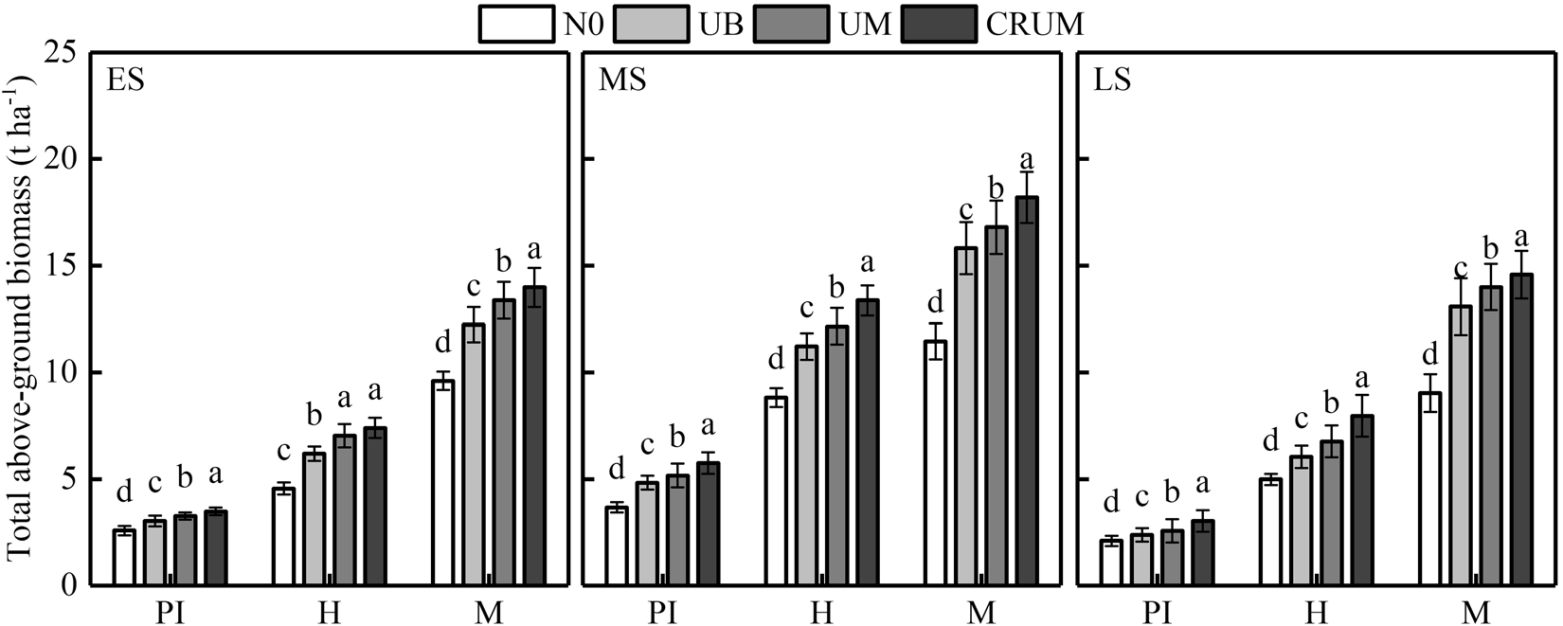
Effects of mechanized side deep nitrogen fertilization on total above-ground biomass of rice in early, middle, and late seasons. ES, MS and LS are early, middle and late season, respectively. PI, H and M are panicle initiation stage, heading stage and maturity stage, respectively. Values are means ± SE of three plots (three replicates). Within the same stage of a cultivar, different letters on columns indicate significant difference (*p* < 0.05, LSD multiple test).

The total above-ground biomass was affected by N application methods and N-fertilizer types (Fig. 4). Total above-ground biomass was lower in the early and late seasons than in the middle season. At the panicle initiation stage, the total above-ground biomass of Yongxian15 and Yongyou1540 was lowest and highest in N0 and CRUM, respectively; and slightly lower in UM and UB than that in CRUM. At the heading stage, the total above-ground biomass of Yongxian15 in the early season and Yongyou1540 in the middle and late seasons was lowest in N0, highest in CRUM; and significantly different between UB and UM. At the maturity stage, total above-ground biomass was highest in CRUM. The treatments could be ranked, from highest total above-ground biomass to lowest, as follows: CRUM > UM > UB > N0.

### Total N uptake

**Figure 5:**
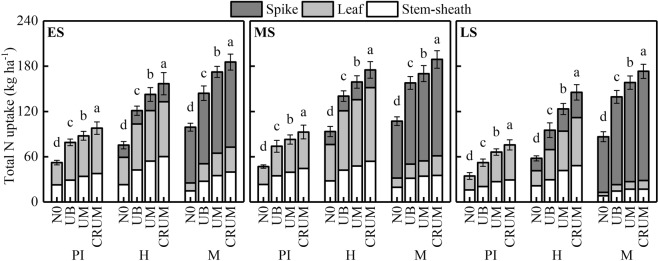
Effects of mechanized side deep nitrogen fertilization on total N uptake of rice in early, middle and late seasons. ES, MS, and LS are early, middle and late season, respectively. PI, H and M are panicle initiation stage, heading stage and maturity stage, respectively. Values are means ± SE of three plots (three replicates). Within the same stage of a cultivar, different letters on columns indicate significant difference in total N uptake (*p* < 0.05, LSD multiple test).

The total N uptake at the panicle initiation, heading and maturity stages are shown in Fig. 5. The highest total N uptake of Yongxian15 and Yongyou1540 was in CRUM at all growth stages, followed by UM and UB. The treatments could be ranked, from highest total N uptake at all growth stages to lowest, as follows: CRUM > UM > UB > N0. In addition, at the maturity stage, more than 60.88% of N was allocated in the panicles, followed by the stem-sheaths and leaves.

### Ammonium (${{\rm{NH}}}_{4}^{+}$-), nitrate (${{\rm{NO}}}_{3}^{-}$-), nitrite (${{\rm{NO}}}_{2}^{-}$-) N concentration in soil

**Figure 6:**
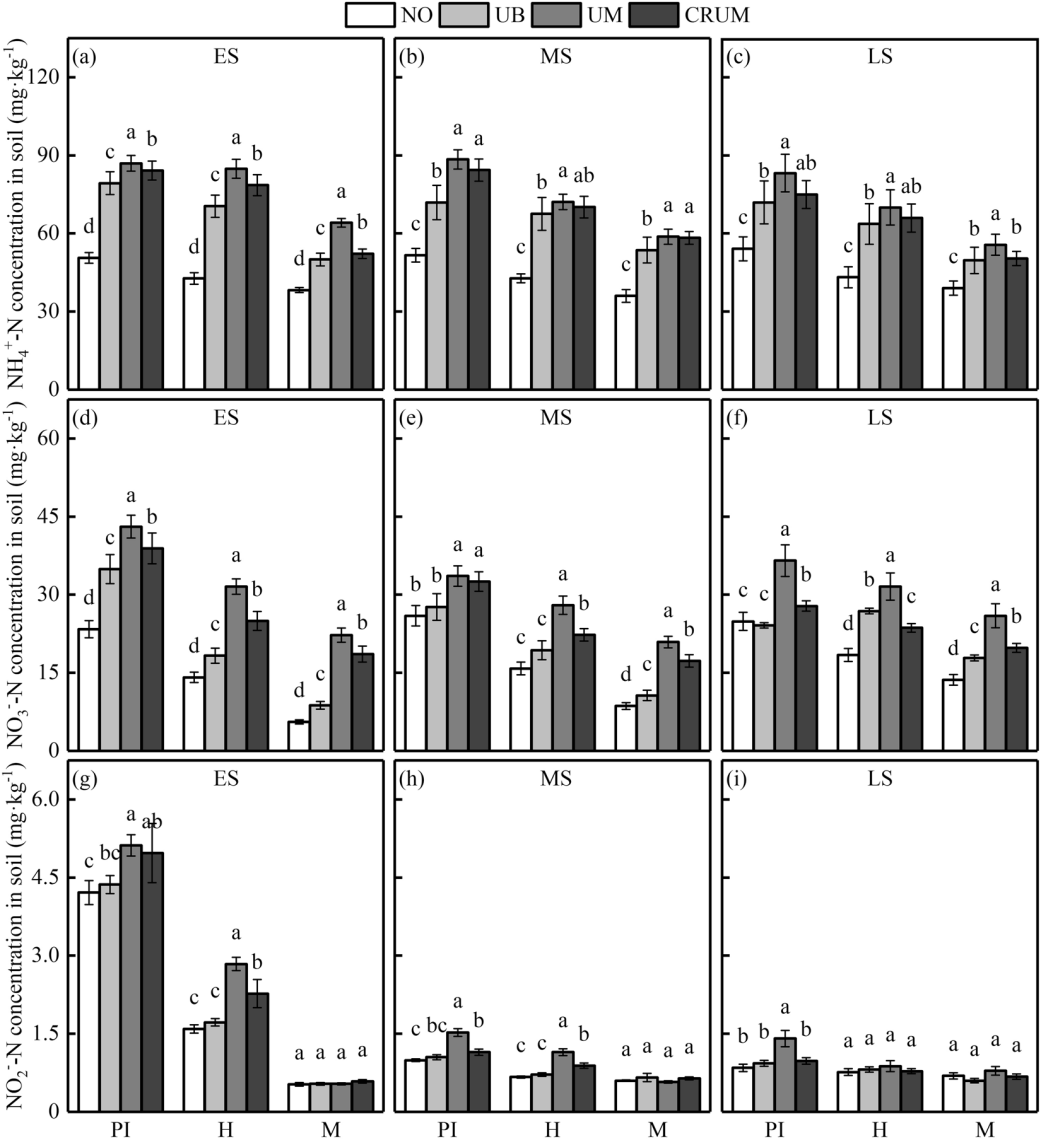
Effects of mechanized side deep nitrogen fertilization on ${{\rm{NH}}}_{4}^{+}$-, ${{\rm{NO}}}_{3}^{-}$-, ${{\rm{NO}}}_{2}^{-}$-N concentration in soil (0–25 cm) in early, middle and late seasons. ES, MS and LS are early, middle and late season, respectively. PI, H and M are panicle initiation, heading and maturity stages, respectively. Values are means ± SE of three plots (three replicates). Within the same stage of a cultivar, different letters on columns indicate significant difference among N application treatments in a season (*p* < 0.05, LSD multiple test).

Each of the treatments had almost the same effects on ${{\rm{NH}}}_{4}^{+}$-, ${{\rm{NO}}}_{3}^{-}$-, ${{\rm{NO}}}_{2}^{-}$-N concentrations in the three seasons (Fig. 6a–i). The average ${{\rm{NH}}}_{4}^{+}$-N concentration ranged from 38.25 (N0) to 87.42 (UM) mg·kg^−1^ in the early season, from 36.03 (N0) to 88.44 (UM) mg·kg^−1^ in the middle season, and from 38.97 (N0) to 82.34 (UM) mg·kg^−1^ in the late season. The ${{\rm{NH}}}_{4}^{+}$-N concentration was highest in UM followed by the CRUM and UB at the panicle initiation, heading and maturity stages in all three seasons, and was lowest in N0. The ${{\rm{NO}}}_{3}^{-}$-N concentration was lowest in N0 (5.56–23.34 mg·kg^−1^ in the early season, 8.63–25.96 mg·kg^−1^ in the middle season, and 13.66–24.88 mg·kg^−1^ in the late season) and highest in UM (22.25–43.06 mg·kg^−1^ in the early season, 20.92–33.59 mg·kg^−1^ in the middle season, and 25.95–36.54 mg·kg^−1^ in the late season). The ${{\rm{NO}}}_{2}^{-}$-N concentration was highest in UM, followed by CRUM and UB, at the panicle initiation stage, heading stage and maturity stage in all growing seasons, and was lowest in N0. In all three seasons, the ${{\rm{NH}}}_{4}^{+}$-, ${{\rm{NO}}}_{3}^{-}$-, and ${{\rm{NO}}}_{2}^{-}$-N concentrations in soil were highest at the panicle initiation stage and lowest at maturity.

### Nitrogen use efficiency

The N dry matter production efficiency (NDMPE), N grain production efficiency (NGPE), N recovery efficiency (NRE), N agronomic use efficiency (NAE), and N physiological efficiency (NPE) of Yongxian15 and Yongyou1540 were affected by N application methods and N fertilizer types in all three seasons (Table 2). The NDMPE of both cultivars was lowest in CRUM, followed by UM and UB. However, there was no significant difference in NDMPE between UM and UB in the middle season. The NGPE differed significantly between N0 and CRUM in the early, middle and late seasons, but not between UM and CRUM in the early and late seasons. The NRE and NAE were highest in CRUM, followed by UM and UB. The NRE and NAE differed significantly between UB and UM (*P* < 0.05) in all seasons. The NPE in all seasons did not differ significantly between UM and CRUM. In the middle and late seasons, the treatments could be ranked, from highest NPE to lowest, as follows: UB > UM > CRUM.

**Table 2:** Effects of mechanized side deep nitrogen fertilization on nitrogen use efficiency of rice in early, middle and late seasons.

Treatments		NDMPE (kg kg^−1^)	NGPE (kg kg^−1^)	NRE (%)	NAE (kg kg^−1^)	NPE (kg kg^−1^)
ES	*Yongxian15*					
	N0	97.16 ± 5.82a	60.26 ± 2.16a			
	UB	85.06 ± 5.08b	50.81 ± 6.99b	25.04 ± 5.40c	7.46 ± 2.55c	31.32 ± 14.42a
	UM	77.66 ± 2.75c	45.06 ± 4.34c	40.69 ± 3.01b	9.97 ± 2.09b	24.76 ± 6.58b
	CRUM	75.35 ± 0.87c	43.96 ± 3.60c	47.97 ± 3.96a	12.17 ± 2.64a	25.43 ± 5.27b
	mean	83.81	50.02	37.90	9.87	27.17
MS	*Yongyou1540*					
	N0	107.08 ± 4.43a	70.56 ± 2.45a			
	UB	100.27 ± 2.76b	66.09 ± 1.57b	21.08 ± 1.52c	11.97 ± 1.28c	56.74 ± 3.13a
	UM	98.80 ± 2.38bc	65.16 ± 2.92b	26.22 ± 0.42b	14.68 ± 1.60b	55.94 ± 5.29ab
	CRUM	96.31 ± 2.64c	61.11 ± 0.72c	34.08 ± 1.53a	16.63 ± 0.23a	48.85 ± 1.53b
	mean	100.62	65.73	27.13	14.43	53.84
LS	*Yongyou1540*					
	N0	104.49 ± 1.36a	71.28 ± 3.55a			
	UB	93.89 ± 1.93b	62.44 ± 1.48b	25.14 ± 3.08c	12.08 ± 0.1c	48.48 ± 5.36a
	UM	88.40 ± 1.08c	59.27 ± 1.47bc	34.15 ± 0.78b	15.32 ± 0.13b	44.86 ± 0.65ab
	CRUM	84.16 ± 1.29d	56.75 ± 1.00c	41.29 ± 1.10a	17.48 ± 0.25a	42.36 ± 0.69b
	mean	92.73	62.43	33.53	14.96	45.23

ES, MS and LS are early, middle and late season, respectively. Values are means ± SE of three plots (three replicates). Different letters indicate significant differences among N application treatments in a season (*p* < 0.05, LSD multiple test).

### Economic profitability

There were significant differences (*P* < 0.05) among the all treatments in fertilizer cost, gross return, net income and benefit-cost ratio (BCR) (Table 3). The fertilizer costs in all three seasons were significantly lower for UB and UM than for CRUM. The highest grain yield was in CRUM, and significantly higher than those in UB and UM. The lowest gross return was in N0, and significantly lower than those in UB, UM and CRUM. In the early, middle and late seasons, the treatments could be ranked, from highest net income and BCR to lowest, as follows: CRUM > UM > UB. In summary, these results demonstrated that manual surface broadcasting of urea is not an appropriate and/or an economical fertilization technology for mechanically transplanted rice.

**Table 3:** Production cost, gross return, and benefit-cost ratio in early, middle, and late seasons.

Treatments		Fertilizer cost (US$ ha^−1^)	Fertilization cost (US$ ha^−1^)	Other input (US$ ha^−1^)	Grain yield (t ha^−1^)	Price (US$ kg^−1^)	Gross return (US$ ha^−1^)	Net income (US$ ha^−1^)	Benefit-cost ratio
ES	Yongxian15								
	N0	158.10	34.58	913.35	5.96 ± 0.24d	0.49	2921.3 ± 119.3c	1815.3 ± 119.3c	2.64 ± 0.11b
	UB	293.42	69.17	913.35	7.30 ± 0.51c	0.49	3579.3 ± 252.0b	2303.4 ± 252.0b	2.81 ± 0.20b
	UM	293.42	57.64	913.35	7.76 ± 0.43b	0.49	3801.0 ± 210.6ab	2536.6 ± 210.4a	3.01 ± 0.17a
	CRUM	331.01	57.64	913.35	8.15 ± 0.58a	0.49	3994.7 ± 285.7a	2692.7 ± 285.7a	3.07 ± 0.22a
	mean	268.99	54.76	913.35	7.29	0.49	3574.1	2337.0	2.88
MS	Yongyou1540								
	N0	158.10	34.58	1348.72	7.56 ± 0.15d	0.48	3626.5 ± 74.0d	2085.2 ± 74.0c	2.35 ± 0.05c
	UB	338.53	69.17	1348.72	10.43 ± 0.31c	0.48	5006.0 ± 149.9c	3249.6 ± 149.9b	2.85 ± 0.09b
	UM	338.53	57.64	1348.72	11.08 ± 0.40b	0.48	5318.2 ± 191.3b	3573.3 ± 191.3a	3.05 ± 0.11a
	CRUM	388.65	57.64	1348.72	11.75 ± 0.28a	0.48	5638.4 ± 133.4a	3843.4 ± 133.4a	3.14 ± 0.07a
	mean	305.95	54.76	1348.72	10.20	0.48	4897.3	3187.9	2.85
LS	Yongyou1540								
	N0	158.10	34.58	1332.58	6.16 ± 0.07d	0.48	2956.3 ± 31.7d	1431.0 ± 31.7d	1.94 ± 0.02c
	UB	315.98	69.17	1332.58	8.69 ± 0.07c	0.48	4173.5 ± 35.3c	2455.8 ± 35.3c	2.43 ± 0.02b
	UM	315.98	57.64	1332.58	9.38 ± 0.07b	0.48	4500.4 ± 32.6b	2794.2 ± 32.6b	2.64 ± 0.02a
	CRUM	359.83	57.64	1332.58	9.83 ± 0.12a	0.48	4718.7 ± 56.4a	2968.6 ± 56.4a	2.70 ± 0.03a
	mean	287.47	54.76	1332.58	8.52	0.48	4087.2	2412.4	2.43

ES, MS and LS are early, middle and late season, respectively. Values are means ± SE of three plots (three replicates). Different letters indicate significant differences among N application treatments in a season (*p* < 0.05, LSD multiple test). Seed cost in early, middle and late season was 39.56, 276.66 and 322.77 US$ ha^−1^, respectively. Rotary tillage cost in early, middle and late season was 184.44 US$ ha^−1^. Planting and management cost in early, middle and late season was 124.50, 230.55 and 214.41 US$ ha^−1^, respectively. Farm pesticides cost in early, middle and late season was 149.86, 242.08 and 195.97 US$ ha^−1^, respectively. Harvest, transportation and drying cost in early, middle and late season was 414.99 US$ ha^−1^.

## Discussion

The grain yield and biomass at maturity stage of Yongyou1540 in the middle and late seasons than Yongxian15 in the early season were much higher because of the longer growth stage and larger potential yields. However, the treatments had similar effects in the three seasons (Table 1 and Fig. 4). Grain yield was much higher in UM and CRUM than in UB in the three seasons. The side deep applicator used in our study (Fig. 1a) opened a slit, uniformly placed N fertilizer at 5.5 ± 0.5 cm soil depth (the anaerobic zone), and then immediately covered it with soil (Fig. 1c). This efficiently provided a sustained N supply to improve growth and photosynthesis characteristics of rice plants for the whole growth period^38,39^ because more ${{\rm{NH}}}_{4}^{+}$-N from urea was retained in soil for a longer time^36,39,40^, thus improving the N uptake and grain yield of the rice plants. The N-fertilizer treatments had significant effects on the number of productive panicles, grain number per panicle, total above-ground biomass and yield. Compared with UB, UM increased yield in the early, middle, and late seasons by 6.30%, 6.23%, and 7.94%, respectively, because of the higher number of productive panicles and grain number per panicle, consistent with the results of previous studies^14,37,40^.

Our results also showed that the grain yield was higher in CRUM than in UM in the three seasons. Compared with UM, CRUM resulted in a much higher grain number per panicle in the early season and much higher number of productive panicles in the middle and late seasons. This finding was not consistent with those of a previous study^41^. The mechanical placement of U and CRU at 5.5 ± 0.5 cm soil depth beside seedlings would inevitably create a special root microenvironment zone because of the longer persistence of ${{\rm{NH}}}_{4}^{+}$-N in the soil^31,42,43^. Compared to deep placement of U, CRU did not significantly increase the number of productive panicles, because multiple seedlings in each hole produced the rapid tiller growth during the early growth stage of the densely distributed seedlings, and the N demands of rice after the heading stage were fully met by the continuous supply of N from CRU, resulting in increased grain number per panicle in the early season.

Our results also demonstrated that total above-ground biomass during the whole growth stage and LAI at heading stage and maturity stage, and the flag leaf *P*n at the heading stage, were much higher in CRUM than in UB (Figs 2, 3 and 4), consistent with previously reported results^37^. The increase in total above-ground biomass in UM and CRUM was mainly related to grain, while these treatments did not have a higher harvest index than that in UB. In a previous study, CRU was shown to promote rice root growth, *P*n, and dry matter accumulation, prolong the leaf functional period, postpone leaf senescence, and enhance grain yield^20,27,28,41^.

Nitrate and nitrite N concentrations at the 0–25 cm soil depth in N application treatments were much higher at panicle initiation in the early season than in the middle and late seasons, and their trends among treatments were almost the same in the three seasons (Fig. 6). At the panicle initiation, heading, and maturity stages, ${{\rm{NH}}}_{4}^{+}$-N concentrations in soil were higher in UM and CRUM than in UB. The ${{\rm{NO}}}_{3}^{-}-$N concentrations in soil at the heading and maturity stages were much lower in UB and CRUM than in UM, indicating that less N was lost by leaching after deep application of CRU^44^. The ${{\rm{NH}}}_{4}^{+}$-N content in soil is considered to be the most important parameter that determines the N uptake of rice, and closely related to NH_3_ volatilization from standing water (more specifically ${{\rm{NH}}}_{4}^{+}$-N in top surface soil), according to previous studies^31,43,44^. The relatively lower concentration of ${{\rm{NH}}}_{4}^{+}$-N in soil was enough to satisfy the N demands of rice plant growth before rapid tillering, but a large amount was absorbed during the active tillering stage and lost through NH_3_ volatilization. Ultimately, CRUM was better in terms of energy saving and the environment. The ${{\rm{NO}}}_{3}^{-}$-N concentration in soil affects the absorption of ${{\rm{NO}}}_{3}^{-}$-N by roots, but a higher concentration results in greater N leaching. In all three seasons, the ${{\rm{NH}}}_{4}^{+}$-N and ${{\rm{NO}}}_{3}^{-}$-N concentrations in soil at 0–25 cm depth were highest in UM, followed by CRUM and UB. The results implied that N losses by leaching and NH_3_ volatilization were higher in the early season than in the middle and late seasons. After U mechanically placed deep in the soil or manually broadcasted into surface water in paddy soil, it is quickly dissolved and hydrolysed into ${{\rm{NH}}}_{4}^{+}$-N by ureases. However, the dissolution rate of CRU is slower because the nutrients are coated with special biological materials with uniform small holes. The urease activity was lower in UM and CRUM than in UB through the whole late growth period, which prolonged N availability to rice roots. These results, which will be reported in detail in another study, are consistent with those of previous studies^42^. The deep placement of N fertilizer obviously decreased nitrate reductase and nitrite reductase in 0–25 cm soil, which weakened the conversion of ${{\rm{NH}}}_{4}^{+}$- into ${{\rm{NO}}}_{3}^{-}$- or ${{\rm{NO}}}_{2}^{-}$-. The ${{\rm{NH}}}_{4}^{+}$-, ${{\rm{NO}}}_{3}^{-}$-, ${{\rm{NO}}}_{2}^{-}$-N concentrations in soil and related enzyme activity in soil showed little difference among UB, UM, and CRUM at the harvest stage, consistent with the results of another study^14,31,43^.

The root length of rice seedlings at transplanting usually 5–8 cm, and the roots extend throughout the 15–20 cm soil layer by about 30 days after transplanting. The added N from urea applied onto the soil surface migrates about 7 cm^45^. By inference, N in our experimental field would rapidly extend 12.5 cm (5.5 + 7 cm) vertically and horizontally in 30 days. In the 15 days after transplanting, rice plants need a little N, and so less N should be allocated at this stage^46^; in the middle growth stage, a large amount of N nutrition is supplied to rice to ensure adequate N uptake and dry matter production as the fertilizer diffuses and the root system extends. In subsequent growth stages, mineral N availability would fully satisfy the growth demands of rice. The total N uptake was higher in the middle season than in the early and late seasons because of the differences in growth and variety characteristics of Yongxian15 and Yongyou1540 (Fig. 5). In all three seasons, deep placement of N fertilizer (CRUM and UM) significantly increased N uptake across all growth stages and increased the NRE, compared with those in UB. This implied that N losses were lower in CRUM and UM than in UB. The deep application of N fertilizer (UM and CRUM) increased N uptake by: (1) reducing N_2_O emission^47^ and NH_3_ volatilization^14^; (2) decreasing surface runoff^31,43^; (3) increasing the ${{\rm{NH}}}_{4}^{+}$-N and ${{\rm{NO}}}_{3}^{-}$-N concentrations in soil^31,42,43^; and (4) synchronizing N availability with plant N demands. The N uptake from the panicle initiation stage to the maturity stage was significantly higher in the mechanically applied N fertilizer treatments (irrespective combinations of N fertilizer applied) than in the manual surface broadcasting treatment.

The N nutrition of rice plants depends on the physical, chemical, and biochemical transformations of N, and on the diffusion dynamics and the forms of N derived from fertilizer and other N sources in soil. The extension and development of the rice root system also affect the N nutrition of rice plants. The NRE and NAE were higher in CRUM and UM than in UB in the early, middle, and late seasons, consistent with previous findings^34,37,38,40,48^. In this study, the deep placement of N fertilizer greatly increased NAE and NRE, regardless of the type of N fertilizer applied (Table 2). In previous studies, the NUE was found to be affected by the N type and the type of pellets used^20,37,41,44^. The higher NRE and NAE under UM were attributed to greater N uptake that was mainly allocated to rice grains. The NRE and NAE were higher in CRUM than in UM in all three seasons, consistent with previously reported results^44^. The N uptake of rice in UM and CRUM were enhanced because of the higher ${{\rm{NH}}}_{4}^{+}$-N and ${{\rm{NO}}}_{3}^{-}$-N concentrations in soil (especially closer to the roots) during the whole rice growth period. Our results also showed that greater pre-heading dry matter accumulation and N uptake were essential to enhance grain yield and NUE. This is because the increased N uptake before the heading stage in UM and CRUM improved N translocation and dry matter production during the grain filling stage, as reported in another study.

Compared with manual surface broadcasting, the mechanized side deep fertilization method had much lower fertilization costs, regardless of the type of fertilizer used (Table 3). Thus, the fertilizer application method was the major cost factor among the inputs. Great savings in labour costs could be made by simultaneously transplanting and fertilizing rice because of the high operational efficiency of this method^33,37,49^. It was reported that in various locations and crop production seasons, the lack of available labour for manual surface broadcasting of fertilizer could result in a crisis^5,7,50^. The rapid economic development in China has meant that the rural labour force has become much smaller as workers have moved to developed coastal areas or cities. Therefore, the rural labour force is becoming scarcer and more expensive. To address this issue, the mechanical side deep fertilization of mechanically transplanted rice can be up-scaled in rice production systems.

In our study, the grain yield in the early, middle and late seasons was highest in CRUM, followed by UM and UB. The BCR and net income were highest in CRUM, followed by UM, and lowest in UB. The combination of higher gross return and lower fertilization cost in CRUM resulted in higher BCR and net income, consistent with previous results^37,42^. In those studies, grain yield and net economic return were increased by deep fertilization of either urea briquettes or N-P-K briquettes compared with broadcasting prilled (pelletized) urea, while deep fertilization of either urea briquettes or N-P-K briquettes reduced N fertilizer use and increased rice production. Other studies have shown that deep placement of fertilizer can increase nutrient-use efficiency^51,52^, thereby reducing the fertilizer requirements without yield penalties. In those studies, basal N fertilizer (40–60% N) was applied by a deep mechanical method or by manual broadcasting, and remaining fertilizer was applied by manual broadcasting. The urea briquette applicator used at the first top and second top dressings resulted in a 42.8% time saving over hand application^42^, which significantly reduced operation times and management costs. Considering that the CRUM method in this study required only two fertilizer applications, increased N availability in paddy fields, and increased grain yields and NUE (Tables 1 and 2), local farmers should be encouraged to adopt this technology. Further research is required to determine whether there are additional benefits of supplying all primary nutrients and balancing fertilization, and to determine whether the mechanized single-dose deep application of a controlled release compound fertilizer (N-P-K) would result in lower labour costs and better nutrient-use efficiency.

## Conclusion

Compared with manual surface broadcasting of N fertilizer, mechanized side deep N fertilization significantly increased the NUE and grain yield of machine-transplanted rice. After the heading stage, the *P*n, total biomass, total N uptake of rice plants were higher in the treatments with mechanically applied fertilizer than in the treatments with manually broadcasted urea. Mechanized side deep fertilization of U and CRU (1:1) also resulted in the highest BCR because of higher total returns and the lower fertilization costs. These results show that mechanized deep side fertilization is an effective technology not only to reduce labour costs and nutrient losses, but also to increase economic profitability. Continuing advancements in such fertilization and production technologies for controlled-release compound fertilizer have the potential to greatly reduce the amount of chemical fertilizer and labour costs, and improve grain yields and resource use efficiency for rice growers worldwide.

## Materials and Methods

### Side deep fertilizer applicator

The machine for the wide-narrow row transplanting of rice accompanied by side deep fertilization was developed by the Jinhe Agricultural Science and Technology Co., Ltd. (Zhejiang, China) (Fig. 1a).

### Experimental site and weather conditions

The field experiments were conducted in three growing seasons: the early season from March to July; the middle season from April to October; and the late season from July to November. All field experiments were conducted in 2017 at the Experimental Research Farm, Langxin Food Professional Cooperatives, Langya Town, Jinhua City, Zhejiang Province, China (119.47°E, 29.02°N, elevation 81 m above sea level). In general, this region has a monsoon and sub-tropical climate (for details, see Table 4). The physicochemical properties of the soil before starting the experiments are shown in Table 5. Soil pH was measured in 1:2.5 (v/v) soil to water ratio using a pH meter. Soil total N, total P, and total K contents were measured by the Kjeldahl method, colorimetric analysis, and flame photometry, respectively. Soil organic matter was determined by the wet combustion method.

**Table 4:** Key meteorological data for rice growing seasons in 2017.

Month	Air temperature (°C)	Minimum temperature (°C)	Maximum temperature (°C)	Average humidity (%)	Precipitation (cm)	Sunshine hours (h)
March	12.01	3.30	23.20	74.77	160.60	94.50
April	19.09	8.30	31.70	69.17	158.61	172.50
May	23.94	16.70	34.60	68.32	41.32	188.80
June	23.88	17.10	35.00	89.90	501.63	62.00
July	31.65	23.80	41.10	64.94	41.32	273.00
August	30.95	24.10	39.10	69.26	48.02	250.10
September	27.03	18.20	38.20	73.17	66.61	157.70
October	20.51	8.30	36.60	73.10	37.62	146.30
November	14.21	6.10	26.10	78.87	138.61	95.80

**Table 5:** Physicochemical properties of soil in early season, middle season, and late season of 2017.

Item	Early rice season	Middle rice season	Late rice season
Soil property	sandy loam	sandy loam	sandy loam
pH-H_2_0 (2.5:1)	5.29	5.60	5.36
Organic carbon (g kg^−1^)	35.04	26.33	33.13
Total N (%)	0.18	0.17	0.17
Total P (%)	0.07	0.08	0.06
Total K (%)	0.58	0.71	0.42
Available N (mg kg^−1^)	62.55	65.66	78.35
Available P-Olsen (mg kg^−1^)	12.76	16.25	18.63
Exchangeable K (mg kg^−1^)	85.50	76.36	88.28

### Experimental material

We used two types of widely grown rice cultivars, Yongxian15 (*Indica* conventional rice) and Yongyou1540 (*Indica/Japonica* hybrid rice), which were supplied by the Ningbo Academy of Agricultural Sciences, Zhejiang, China and Ningbo Seed Co., Ltd., China, respectively. Yongxian15 (early season rice) had a growth period of 107.9 days. Yongyou1540 had growth periods of 151.5 and 123.9 days as middle-season and late-season rice, respectively. The two types of N fertilizers were common urea (U, total N content TN = 46.7%) and controlled release urea (CRU, total N content TN = 41.6%, with a slow release period of 60 days), both of which were manufactured by Sinofert Holdings Limited, China.

### Experimental design and treatments

Two fertilization methods were used: manual surface broadcast (B), and 5.5 ± 0.5 cm-depth and horizontal width mechanized side deep placement (M). Four N-fertilizer treatments were arranged in a randomized complete block design with three replications in each season. The experimental plots were 120 m^2^ in the early rice season and 100 m^2^ in the middle and late rice seasons. The N-fertilizer treatments were as follows:

#### Early rice season

UB: 144 kg N ha^−1^ as U was broadcasted manually onto the soil surface as seedling fertilizer, and 36 kg N ha^−1^ as U was broadcasted manually onto the soil surface at the panicle initiation stage.

UM: 144 kg N ha^−1^ as U was mechanically fertilized at 5.5 ± 0.5 cm soil depth beside seedlings when transplanting, and 36 kg N ha^−1^ as U was broadcasted manually onto the soil surface at the panicle initiation stage.

CRUM: 72 kg N ha^−1^ as U and 72 kg N ha^−1^ as CRU were mechanically fertilized at 5.5 ± 0.5 cm soil depth beside seedlings when transplanting, and 36 kg N ha^−1^ as U was broadcasted manually onto the soil surface at the panicle initiation stage.

#### Middle rice season

UB: 192 kg N ha^−1^ as U was broadcasted manually onto the soil surface as seedling fertilizer, and 48 kg N ha^−1^ as U was broadcasted manually onto the soil surface at the panicle initiation stage.

UM: 192 kg N ha^−1^ as U was mechanically fertilized at 5.5 ± 0.5 cm soil depth beside seedlings when transplanting, and 48 kg N ha^−1^ as U was broadcasted manually onto the soil surface at the panicle initiation stage.

CRUM:96 kg N ha^−1^ as U and 96 kg N ha^−1^ as CRU were mechanically fertilized at 5.5 ± 0.5 cm soil depth beside seedlings when transplanting, and 48 kg N ha^−1^ as U was broadcasted manually onto the soil surface at the panicle initiation stage.

#### Late rice season

UB: 168 kg N ha^−1^ as U was broadcasted manually onto the soil surface as seedling fertilizer, and 42 kg N ha^−1^ as U was broadcasted manually onto the soil surface at the panicle initiation stage.

UM: 168 kg N ha^−1^ as U was mechanically fertilized at 5.5 ± 0.5 cm soil depth beside seedlings when transplanting, and 42 kg N ha^−1^ as U was broadcasted manually onto the soil surface at the panicle initiation stage.

CRUM: 84 kg N ha^−1^ as U and 84 kg N ha^−1^ as CRU were mechanically fertilized at 5.5 ± 0.5 cm soil depth beside seedlings when transplanting, and 42 kg N ha^−1^ as U was broadcasted manually onto the soil surface at the panicle initiation stage.

Plots with no added N fertilizer were established as the blank control (N0) in each season. Each treatment received 90 kg P_2_O_5_ ha^−1^ as superphosphate and 120 kg K_2_O ha^−1^ as potassium chloride. All phosphate fertilizer and 50% of potash fertilizer were broadcasted onto the soil surface 1 day before transplanting, and 50% of potash fertilizer was applied at the panicle initiation stage in all treatments. In all rice seasons, the strategy for water management was the sequence of flooding, midseason drainage, re-flooding, moist intermittent irrigation, and drainage. Weeds, insects, and diseases were intensively controlled by chemicals. Other field practices, such as field preparation, tillage, and puddling, were carried out manually according to the local farming practices.

### Total above-ground biomass and leaf area index (LAI)

Six hills of plants were sampled from each plot according to average tiller number at the panicle initiation, heading and maturity stages. The adhered soil was thoroughly washed from the plants, and then the panicles, leaves, and stems with leaf sheaths were cut away from the plants after the heading stage. A LI-3100C Area Meter (LI-COR, Inc., USA) was used to measure the leaf area of each green leaf, then the leaf area per square meter was calculated as LAI. To record the total above-ground biomass, the sampled plants were dried at 105 °C for 30 min to halt biological activity, and then dried at 80 °C to constant weight (DHG-9625A, Shanghai Yiheng Scientific Instruments Co., Ltd., Shanghai, China).

### Net photosynthetic rate (*P*n)

At the heading stage, LI-6400XT Portable Photosynthesis System (LI-COR, Inc., Lincoln, NE USA) was used to measure *P*n of the flag leaves in the early, middle, and late seasons in 2017. The photosynthetically active radiation was controlled at 1200 μmol m^−2^ s^−1^ provided by a 6400-2B LED light source. Six representative flag leaves from each plot were measured and recorded, and the mean values were calculated for *P*n.

### Yield and its components

At the maturity stage, six hills of plants were collected from each plot to measure yield components. The straw and panicles were cut away from the plants. To separate filled grains, each grain was separated from the rachides of the spike (through manual threshing) and immersed in distilled water. Those that floated were considered to be unfilled grains. To measure the gross number of spikelets, we counted spikelets in three representative subsamples of 30 g. The average weight of half-filled spikelets was determined. The grain number per panicle, the percentage of grain filling, and the 1000-grain-weight were measured according to Pan S. *et al*.^37^. Rice plants from a 60.0 m^2^ area were harvested in plots and the grain yield was calculated based on a standardized moisture content of 14%.

### Plant N content, N uptake, and N use efficiency

At the panicle initiation, heading and maturity stages, we collected panicles, leaves, stems with leaf sheath samples from each plot to analyse their total N contents. The plant samples (0.20 g) were digested for 2 h in H_2_SO_4_-H_2_O_2_ solution at 420 °C and analysed by the micro-Kjeldahl method (Kjeltec^TM^ 8400, FOSS, Denmark).

N uptake was calculated using the formula TDM × NC, where TDM represents the total dry matter of panicles, leaves, and stems with leaf sheaths, and NC represents the N concentration in panicles, leaves, and stems with leaf sheaths.

Aspects of N use efficiency, such as N dry matter production efficiency (NDMPE), N grain production efficiency (NGPE), N recovery efficiency (NRE), N agronomic use efficiency (NAE), and N physiological efficiency (NPE) were calculated as the following formulas:

$${\rm{NDMPE}}={\rm{TBup}}/{\rm{TNup}}$$

$${\rm{NGPE}}={\rm{GY}}/{\rm{Nup}}$$

$${\rm{NRE}}=({\rm{Nup}}-{\rm{N0up}})/{\rm{FN}}$$

$${\rm{NAE}}=({\rm{GY}}-{\rm{GY0}})/{\rm{FN}}$$

$${\rm{NPE}}=({\rm{GY}}-{\rm{GY0}})/({\rm{Nup}}-{\rm{N0up}})$$

where TBup and TNup denote the total above-ground biomass and total N uptake above-ground, respectively; GY and GY0 represent grain yields in N-fertilized plots and N0 plots, respectively; Nup and N0up denote total N uptake above-ground in N-fertilized plots and N0 plots, respectively; FN denote the total N application rate in N-fertilized plots.

### Soil ammonium (${{\rm{NH}}}_{4}^{+}$-), nitrate (${{\rm{NO}}}_{3}^{-}$-) nitrite (${{\rm{NO}}}_{2}^{-}$-) N concentration

Six soil samples (0–25 cm depths) were collected from each plot using a soil sampler with a diameter of 5 cm and mixed to make one composite soil sample, at panicle initiation stage (One day before fertilization), heading stage and maturity stage (One day after harvest) in the early, middle and late seasons. Fresh soil samples were kept for the determination of ${{\rm{NO}}}_{3}^{-}$-N concentration. Fresh soil samples were kept at −20 °C for the determination of ${{\rm{NO}}}_{2}^{-}$-N concentration. Soil samples were air dried to a constant weight and ground to pass through 0.149 mm sieve for the determination of ${{\rm{NH}}}_{4}^{+}$-N concentration. Soil ${{\rm{NO}}}_{3}^{-}$-N, ${{\rm{NO}}}_{2}^{-}$-N, and ${{\rm{NH}}}_{4}^{+}$-N concentration was determined by the trans-nitration of salicylic acid method, the diazotization-coupling spectrophotometric method with N-(1-naphthyl) ethylenediamine as the coupling agent and the indophenol blue method, respectively.

### Economic profitability

Economic profitability was calculated from the rice production costs and the net income obtained from the rice sale price. The rice production costs consisted of fertilizer cost, fertilization cost, other input. Fertilizer cost included the expenditures on N fertilizer, phosphate fertilizer, potash fertilizer. Fertilization cost included the expenditures on labour and fuel. Other input included the expenditures on seed, rotary tillage, planting and management (labour and fuel on raising rice seedlings, transplanting, spraying pesticides, and water management), farm pesticides, harvest, transportation, and drying. All of these costs were the local average price in the rice growing seasons. Net income was estimated by subtracting various costs and expenses from the calculated gross return. The ratio of gross return to total production cost was calculated as the benefit cost ratio.

### Data analysis

Data are presented as means ± SE of three plots (three replicates). The dataset for early, middle, and late season were analysed using SAS® 9.1. Pairwise means comparison among treatments was conducted with least significant difference (LSD) tests at the 0.05 level of probability.

## Data Availability

The data used or analysed during the current study are available from the corresponding author on reasonable request.
